# Flexibility in Red Sea *Tridacna maxima*‐Symbiodiniaceae associations supports environmental niche adaptation

**DOI:** 10.1002/ece3.7299

**Published:** 2021-03-11

**Authors:** Susann Rossbach, Benjamin C. C. Hume, Anny Cárdenas, Gabriela Perna, Christian R. Voolstra, Carlos M. Duarte

**Affiliations:** ^1^ Biological and Environmental Science and Engineering Division Red Sea Research Center (RSRC) and Computational Bioscience Research Center (CBRC) King Abdullah University of Science and Technology (KAUST) Thuwal Saudi Arabia; ^2^ Biological and Environmental Science and Engineering Division Red Sea Research Center (RSRC) King Abdullah University of Science and Technology (KAUST) Thuwal Saudi Arabia; ^3^ Department of Biology University of Konstanz Konstanz Germany

**Keywords:** ITS2, Red Sea, Symbiodiniaceae, SymPortal, *Tridacna maxima*, Tridacninae

## Abstract

Giant clams (Tridacninae) are important members of Indo‐Pacific coral reefs and among the few bivalve groups that live in symbiosis with unicellular algae (Symbiodiniaceae). Despite the importance of these endosymbiotic dinoflagellates for clam ecology, the diversity and specificity of these associations remain relatively poorly studied, especially in the Red Sea. Here, we used the internal transcribed spacer 2 (ITS2) rDNA gene region to investigate Symbiodiniaceae communities associated with Red Sea *Tridacna maxima* clams. We sampled five sites spanning 1,300 km (10° of latitude, from the Gulf of Aqaba, 29°N, to the Farasan Banks, 18°N) along the Red Sea's North‐South environmental gradient. We detected a diverse and structured assembly of host‐associated algae with communities demonstrating region and site‐specificity. Specimens from the Gulf of Aqaba harbored three genera of Symbiodiniaceae, *Cladocopium, Durusdinium*, and *Symbiodinium*, while at all other sites clams associated exclusively with algae from the *Symbiodinium* genus. Of these exclusively *Symbiodinium*‐associating sites, the more northern (27° and 22°) and more southern sites (20° and 18°) formed two separate groupings despite site‐specific algal genotypes being resolved at each site. These groupings were congruent with the genetic break seen across multiple marine taxa in the Red Sea at approximately 19°, and along with our documented site‐specificity of algal communities, contrasted the panmictic distribution of the *T. maxima* host. As such, our findings indicate flexibility in *T. maxima*‐Symbiodiniaceae associations that may explain its relatively high environmental plasticity and offers a mechanism for environmental niche adaptation.

## Graphical abstract

Here we demonstrate site‐ and region‐specific structuring of giant clam‐Symbiodiniaceae associations across 10° of latitude in the Red Sea. This structuring indicates a flexibility in clam‐algal associations that may explain their high environmental plasticity and offer a strategy for niche adaptation.

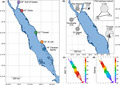

## 1 INTRODUCTION

Giant clams (Tridacninae subfamily) are prominent members of Indo‐Pacific corals reefs, where they play important ecological roles (Neo et al., 2015), by providing a food source for different predators and scavengers (Alcazar, 1986), shelter for commensal organisms (De Grave, 1999), and settling substrate for epibionts (Vicentuan‐Cabaitan et al., 2014). As Tridacninae filter large volumes of water, thereby absorbing dissolved organic carbon (Klumpp & Griffiths, 1994), they are even hypothesized to form a “giant clam loop,” resembling the “sponge loop” (De Goeij et al., 2013; Mies, 2019). Further, giant clams are considered an ecosystem‐engineering species (Neo et al., 2015), as they can form reef‐like structures (Andréfouët et al., 2005; Gilbert et al., 2005). They are also harvested by humans for food and ornamental purposes (Brown & Muskanofola, 1985; Mies et al., 2017).

Tridacninae stand out among other bivalves as they are one of the few molluscan groups that live in a symbiotic relationship with dinoflagellates in the family Symbiodiniaceae (Taylor, 1969; Yonge, 1936). The relationship is comparable to the symbiosis of corals and their associated algae with respect to the symbionts providing a substantial amount of energy in the form of photosynthates for the host. Clam veliger larvae acquire free‐living Symbiodiniaceae from the water column (Fitt & Trench, 1981) and harbor their symbionts extracellularly in a tubular system, that originates from the digestive diverticular ducts of the stomach, extending mainly in the outer mantle (Norton et al., 1992). Although Tridacninae have generally been described as mixotrophic (Hawkins & Klumpp, 1995; Klumpp et al., 1992), this photosymbiosis seems to be obligate for the clam host, as previous studies report that Tridacninae often perish in the absence of their algal symbionts (Addessi, 2001; Leggat et al., 2003), for example, following bleaching (i.e., the expulsion of their symbiotic algae; Glynn, 1993).

A considerable biological diversity exists within symbiotic Symbiodiniaceae taxa (LaJeunesse et al., 2018; Thornhill et al., 2014). Importantly, the physiology of the algal symbionts may modulate the phenotype of their marine invertebrate hosts (Cunning et al., 2015; Howells et al., 2020; Rädecker et al., 2015; Rädecker et al., 2021; Silverstein et al., 2015; Terraneo et al., 2019). As an example, many *Durusdinium* taxa appear to be relatively stress‐tolerant (LaJeunesse et al., 2014), for example, to warm and cold temperature‐induced bleaching (Silverstein et al., 2017), and certain specialist *Cladocopium* taxa are found in the hottest coral‐containing waters on Earth (Hume et al., 2016). Characterization of these algal assemblages, including assessing their potential for change, is therefore of particular importance when considering the adaptive potential of the holobiont (the consideration of the animal host and all associating organisms as a single unit). By virtue of their socio‐economic and ecological value, these associations have received considerable attention in corals (LaJeunesse et al., 2004, 2010; LaJeunesse et al., 2014, 2018; Lewis et al., 2019; Pettay et al., 2015; Sampayo et al., 2008; Stat et al., 2009; Thornhill et al., 2006). In contrast, Tridacninae‐Symbiodiniaceae associations have received limited attention (as reviewed by Mies, 2019).

Among the markers that are available for assessing Symbiodiniaceae diversity, the internal transcribed spacer 2 (ITS2) of the rRNA gene array is most commonly used (Cunning et al., 2017; Hume, D’Angelo et al., 2018; Hume, Ziegler et al., 2018; LaJeunesse, 2002). This marker is considerably multicopy in nature with a single Symbiodiniaceae cell potentially containing hundreds of copies of the gene (Arif et al., 2014; LaJeunesse, 2002; Thornhill et al., 2007). While this intragenomic character may complicate analyses, profiling approaches such as the SymPortal framework (Hume et al., 2019), where sets of sequences may be considered diagnostic of a given genotype, make use of this intragenomic diversity to afford improved resolutions.

Use of the ITS2 marker, and to a lesser extent the full ITS region, is common in the assessment of Tridacninae‐Symbiodiniaceae associations (DeBoer et al., 2012; Lim et al., 2019; Pappas et al., 2017; Weber, 2009). The resolutions achieved in these studies varies according to the specific marker (i.e., ITS2 or full ITS region; Pappas et al., 2017; Weber, 2009), the sequencing technology used (Lim et al., 2019), and the degree to which intragenomic sequence diversity is taken into account (Lim et al., 2019). However, due to analytical (e.g., treatment of all sequence diversity as intergenomic in origin; Lim et al., 2019) and technological (e.g., DGGE; DeBoer et al., 2012) limitations, resolutions in these studies are generally limited to the assessment of the most abundant ITS2 sequence present. This level of resolution masks the majority of ecologically relevant inferences as considerable phenotypic diversity exists between Symbiodiniaceae taxa that share a most abundant ITS2 sequence in common (Arif et al., 2014; Hume et al., 2013; Hume et al., 2015; LaJeunesse et al., 2014; Thornhill et al., 2014).

In general, Tridacninae have been reported to associate with Symbiodiniaceae of three genera (Baillie et al., 2000; Carlos et al., 2000; Ikeda et al., 2017; Lim et al., 2019; Weber, 2009), *Cladocopium*, *Durusdinium,* and *Symbiodinium,* previously known as Clades C, D, and A, respectively (recently revised by LaJeunesse et al., 2018). Tridacninae are also known to be capable of associating with multiple Symbiodiniaceae genera simultaneously (DeBoer et al., 2012; Mies, 2019). Previous work alludes to the specific community composition being dependent on a number of factors including, but not limited to, location, thermal regime, light regime, and host size (Belda‐Baillie et al., 1999; DeBoer et al., 2012; Lim et al., 2019; Mies, 2019).

**FIGURE 1:**
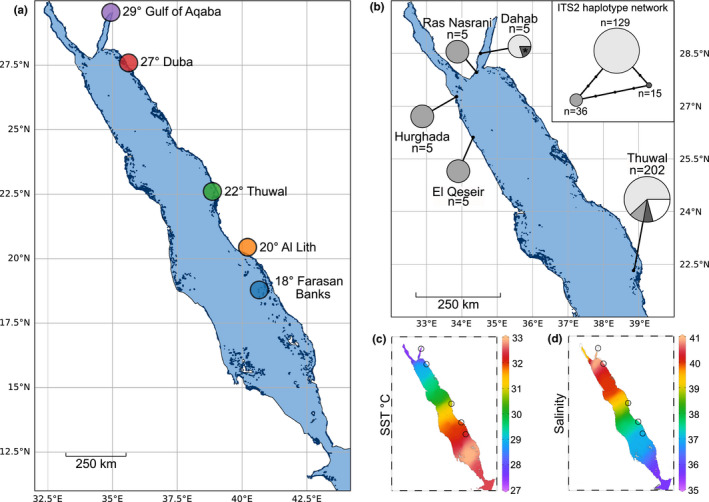
Sampling maps, ITS2 sequence haplotype frequencies, and environmental gradients. (a) The five sampling sites of *Tridacna maxima* for this study along the Saudi Arabian Red Sea coast in the northern (29°—Gulf of Aqaba and 27°—Duba), central (22°—Thuwal), and southern (20°—Al Lith and 18°—Farasan Banks) Red Sea. The site colors correspond to the site colors in Figures 2 and 3. (b) Sampling sites from Weber, 2009 (Dahab, Ras Nasrani, Hurghada and El Qeseir) and Pappas et al. (2017) (9 sampling sites in close proximity on the same reef system referred to as Thuwal). For each sampling location, the number of ITS2 sequences is given as “*n* = x” and the proportion of the sequences that are one of the three most abundant haplotypes (shades of gray) or some other unclassified sequence (white) are detailed as pie charts. The asterisk at the Dahab site refers to the one sequence from the Weber, 2009 study that contained an ambiguous nucleotide in the exact nucleotide position that differentiates the first most abundant haplotype from the third most abundant nucleotide. For the creation of this figure, this sequence was considered to represent the third most abundant haplotype. Shades of gray refer to the inset ITS2 haplotype network. (b‐inset) ITS2 haplotype network. Each node represents a different ITS2 sequence with size proportional to the number of sequences recovered from the Weber and Pappas studies combined. The number of base pairs (bp) different between each of the sequences is denoted by the number of small black nodes on the network edges (one node represents one bp difference). (c) Maximum annual sea surface temperatures (averaged for 1982–2015; from Chaidez et al., 2017). (d) Salinity (from Ngugi et al., 2012). For (a) and (b) reference reefs from the United Nations Environment Programme—World Conservation Monitoring Center's (UNEP‐WCMP) Global Distribution of Coral Reefs dataset (IMaRS‐USF, 2005; Spalding et al., 2001; UNEP‐WCMC & WRI, 2010) are plotted in dark blue using reefMapMaker (Hume & Voolstra, 2021). (b) and (c) were created using Ocean Data Viewer (Schlitzer, 2015; https://odv.awi.de/) and have the five sampling sites of this study overlaid as empty black circles for reference

Characterizations of *Tridacna maxima*‐Symbiodiniaceae associations within the Red Sea are limited to two studies (Pappas et al., 2017; Weber, 2009; hereafter referred to as Weber and Pappas et al., respectively) that cover a limited geographical range. Pappas et al., investigated 207 samples, from nine sites, all located at 22°N at the eastern coast of the central Red Sea and within 28 km of the “Thuwal” site from this study (given their proximity to each other, these nine sites will hereafter be referred to as a single site, “Thuwal”), while Weber analyzed samples from a total of 20 clams, originating from four sites, of which two were located in the Gulf of Aqaba at 27° (Ras Nasrani) and 28°N (Dahab), and two off the Egyptian coast in the North‐western Red Sea at a latitude of 25° (El Qeseir) and 27° (Hurghada), respectively (Figure 1). Given the extraordinary latitudinal hydrographical gradients that exist in the Red Sea (Agulles et al., 2020; Arz et al., 2003; Berumen et al., 2019; Chaidez et al., 2017), the coverage thus far available provides a limited representation of the region. Here we build on these prior characterizations using the ITS2 marker and the SymPortal framework to conduct a fine‐scale characterization of Symbiodiniaceae associations in Red Sea *T. maxima* giant clams across the Red Sea's North‐South gradient (from the Gulf of Aqaba at a latitude of 29°N to the Farasan Banks at 18°N), covering 1,300 km of overwater distance, and environmental differences.

## 2 MATERIAL AND METHODS

### 2.1 Sample collection

Between January 2018 and January 2019, we collected a total of nine *T. maxima* mantle tissue samples, at each of five different reef sites along the Saudi Arabian Red Sea coastline (Figure 1a; 45 samples in total). Samples were collected via SCUBA in water depths between 2 and 5.5 m with sampling sites following a latitudinal gradient covering 10° (Figure 1a). The clams collected had an average length of 15.7 cm + − 0.8 (+‐ *SD*) which corresponds to sexually mature individuals (Manu & Sone, 1995). Sampling sites were located in the North‐eastern Red Sea, that is, Haql in the Gulf of Aqaba at 29° (34.937314 N, 29.259089 E) and Duba at 27° (27.304722 N, 35.622222 E); in the central Red Sea, that is, Rose reef, located close to King Abdullah University of Science and Technology in Thuwal at 22° (22.322222 N, 38.857222 E), and in the South‐eastern Red Sea, that is, at a reef close to Al Lith at 20° (20.158111 N, 40.210603 E) and in the Farasan Banks at 18° (18.503297 N, 40.661747 E) (Figure 1a). In covering a wide range of latitudes along the Red Sea coast, the sampling sites also reflect distinct environmental settings with regard to temperature (Figure 1c) and salinity (Figure 1d), which display pronounced latitudinal gradients in the region that negatively covary (Agulles et al., 2020; Chaidez et al., 2017; Ngugi et al., 2012).

During the sampling, a metal bolt was used to keep the shell valves open and a small piece of mantle tissue of approximately 1 cm^2^ was cut using a scalpel. Samples were then immediately frozen in seawater, using liquid nitrogen, and transported back to the laboratory where they were kept at −80°C until further analysis.

At the two southern Red Sea stations (20° Al Lith and 18° Farasan Banks), seawater samples were collected (one at each site), at the same reefs and depths as the mantle tissue sampling using a 9 L polycarbonate carboy container (Nalgene, Thermo Scientific Fisher). The containers were kept in a cooling box on ice until arrival at the laboratory, where, for each of the two reefs, 2 L of seawater was immediately filtered through a 0.22 µm hydrophilic polyvinylidene fluoride (GVWP) filter (Millipore, Merck KGaA) using a peristaltic pump. Filters were instantly frozen at −80°C for further analysis.

### 2.2 Tissue homogenization and DNA isolation

The frozen *T. maxima* mantle tissue samples were separated from the frozen seawater and homogenized using a Freezer/Mill^®^ (Model 6,875, SPEX^®^ Sample Prep) by grounding up the tissues in liquid nitrogen at a rate of ten impacts per second for a total of 90 s. The Freezer/Mill^®^ PVC tubes were cleaned thoroughly with 10% bleach between samples and the ground tissues were then dissolved in 5 ml Milli‐Q water (sterilized under UV light for 1 hr). The homogenate was transferred to 1.5 ml Eppendorf tubes and samples were frozen at −20°C until further processing.

In total, we extracted DNA from 50 samples, corresponding to 45 *T. maxima* mantle tissue samples, two water samples, and three negative controls: 1 to assess for DNA extraction kit contamination, 1 to assess for PCR reagent contamination, and 1 to assess for carryover contamination during the tissue homogenization. The carryover contamination negative sample was generated by adding Milli‐Q water to the cryotubes after they had been rinsed as part of the rinsing step carried out between each sample's processing. DNA extractions from the tissue samples were extracted with the Qiagen DNeasy 96 Blood & Tissue Kit (Qiagen) following the manufacturer's instructions with minor modifications. Briefly, 90 μl of the tissue homogenate was added to 1.5 ml Eppendorf tubes containing 90 μl of ATL buffer and 20 μl of Proteinase K and incubated in an Eppendorf ThermoMixer at 56°C and 300 rpm for 1 hr. DNA extractions were then continued according to the manufacturer's instructions. DNA concentrations were measured using a Qubit 2.0 fluorometer (ThermoFisher Scientific). Water filters were thawed and placed in 1.5 ml Eppendorf tubes, 360 μl of ATL buffer, and 40 μl Proteinase K buffer were added, and the tubes were incubated at 56°C for 20 min. DNA extractions were then continued according to the manufacturer's instructions using the Qiagen DNeasy 96 Blood & Tissue Kit (Qiagen). DNA concentrations were measured using a Qubit 2.0 fluorometer (ThermoFisher Scientific), and samples were adjusted to 10 ng/μl.

To amplify the ITS2 region, the primers SYM_VAR_5.8S2: 5′ (TCGTCGGCAGCGTCAGATGTGTATAAGAGACAG)GAATTGCAGAACTCCGTGAACC 3′ and SYM_VAR_REV: 5′ (GTCT CGTGGGCTCGGAGATGTGTATAAGAGACAG)CGGGTTCWCTTGTYTGACTTCATGC 3′ (Hume et al., 2013, 2015; Hume, Ziegler et al., 2018) were used (Illumina adaptor overhangs underlined). For all samples, triplicate PCRs were performed using 1 μl of DNA (3–20 ng) using the Qiagen Multiplex PCR kit and a final primer concentration of 0.5 μM in a reaction volume of 10 μl. Thermal cycling conditions were as follows: 95°C for 15 min, followed by 30 cycles of 95°C for 30 s, 56°C for 90 s, 72°C for 30 s, and a final extension cycle of 72°C at 10 min. Five µl of the PCR was run on a 1% agarose gel to visualize amplification.

The PCR negative did not return valid ITS2 sequence reads, and the DNA extraction control returned a greatly reduced number of reads and was dominated by a sequence that was not a defining intragenomic sequence variant (DIV; a sequence that is part of an ITS2 type profile definition; e.g., A1aw, A1em, and A1aw are the three DIVs of the A1‐A1aw‐A1em profile) in any of the predicted ITS2 type profiles (Figure S1). By contrast, the negative control for potential carryover contamination during the tissue homogenization returned a predicted profile that matched the profile most dominant at the Duba (27°) site. However, since this profile was only found in five samples that all originated from Duba, and that other profiles were predicted at this site, it can be assumed that there was no bias introduced from putative contaminants in the analysis.

### 2.3 Sequencing of internal transcribed spacer 2 (ITS2)

Symbiodiniaceae communities were characterized using next‐generation sequencing of the rDNA ITS2 region (ITS2). Triplicates for each sample were pooled, and samples were cleaned using ExoProStar 1‐step (GE Healthcare). Samples were then indexed using the Nextera XT Index Kit v2 (dual indexes and Illumina sequencing adaptors added). Successful addition of indexes was confirmed by comparing the length of the initial PCR product to the corresponding indexed sample on a 1% agarose gel. Samples were then cleaned and normalized using the SequalPrep Normalization Plate Kit (Invitrogen). The ITS2 libraries were pooled in an Eppendorf tube (4 μl per sample) and concentrated using a CentriVap Benchtop Vacuum Concentrator (Labconco). Following this, quality of the library was assessed using the Agilent High Sensitivity DNA Kit on the Agilent 2100 Bioanalyzer (Agilent Technologies). Quantification was done using Qubit (Qubit dsDNA High Sensitivity Assay Kit; Invitrogen). Sequencing was performed at 6 pM with 20% phiX on the Illumina MiSeq platform at 2 × 301 bp paired‐end in the KAUST Bioscience Core Laboratory (BCL). Raw sequencing data are available under NCBI BioProject accession number #657469.

### 2.4 Analyses using SymPortal

Demultiplexed paired fastq.gz sequencing files were submitted directly to the remote instance of SymPortal for analysis (symportal.org; Hume et al., 2019). The SymPortal analytical framework was used to predict ITS2 profiles as proxies of Symbiodiniaceae genotypes and to generate between‐sample dissimilarity metrics, based on ITS2 sequence assemblages. The framework leverages information encoded in the intragenomic sequence diversity, harbored within the Symbiodiniaceae genome, to offer fine‐scale delineations. To plot ITS2 type profile relative abundances, we made use of the ITS2 type profile absolute count table output by SymPortal. As part of the built‐in quality control pipeline of SymPortal, Minimum Entropy Decomposition (MED; Eren et al., 2015; Hume et al., 2019) is performed on a per‐sample basis before the sequence abundances are used to predict ITS2 type profiles. To plot ITS2 relative sequence abundances, we used the post‐MED absolute abundance sequence count tables (as opposed to the pre‐MED sequence count tables). To produce a heat map of between‐site average sample dissimilarities, and two PCoA ordinations, we used the *Symbiodinium,* Bray–Curtis‐derived, between‐sample dissimilarity matrix (including a square root transformation) that is output by default by SymPortal. The between‐site average dissimilarities were computed using a bespoke Python script and plotted using matplotlib's implementation of imshow. Specifically, for a given pairwise site comparison, for every sample in the first site, the distance to every sample in the second site was collected. A mean average and standard deviation were then computed from these distances. For self‐site comparisons (e.g., Duba‐Duba), average within‐site distances were calculated. PCoAs were computed using Python and the scikit implementation of pcoa and plotted using matplotlib's implementation of scatter. To assess for statistical difference between sites across samples based on *Symbiodinium* ITS2 sequence assemblages, we ran a one‐factor PERMANOVA (Anderson, 2001) using scikit‐bio's function PERMANOVA and the *Symbiodinium* Bray–Curtis square root transformed distances. PERMANOVA is resilient to heteroscedasticity across factor groups when factor groups have equal numbers of samples (i.e., a balanced experimental design; Anderson & Walsh, 2013). Given the balanced design in this study, a PERMDISP2 analysis (Anderson, 2006) was not conducted. To further investigate the sequences driving structure in the Bray–Curtis‐derived distances, we conducted a SIMPER analysis (Clarke, 1993) on the post‐MED *Symbiodinium* sequences with site as the grouping factor. The abundances were square‐root transformed (to match the transformation of the abundance matrix used in the Bray–Curtis calculation), and the analysis was conducted in Python using ecopy's simper function.

### 2.5 Reanalysis of ITS1‐5.8S‐ITS2 haplotypes from previous characterizations

Two previous giantclam‐Symbiodiniaceae characterizations exist from the Red Sea (i.e., Weber, 2009 and Pappas et al., 2017). These studies characterized the dominant Symbiodiniaceae genotypes by analyzing the full ITS region (ITS1‐5.8S‐ITS2) of the rRNA array resolved via Sanger sequencing. To assess for similarity in sampled haplotypes between these studies, we collated all sequences of Red Sea origin from them, computed a multiple sequences alignment using MAFFT (Katoh & Standley, 2013), and cropped at the 5′ and 3′ end of the alignment so that, with the exception of three short sequences that were removed from the alignment; all sequences were represented across the full alignment length. For reference, the A1 ITS2 sequence, as defined in the SymPortal remote database (symportal.org) was included in the alignment (independent of any cropping). The full alignment is provided in Dryad submission https://doi.org/10.5061/dryad.k6djh9w50.

### 2.6 Comparison of clam and seawater ITS2 sequence assemblages to support inferences of selectivity

We used the two seawater samples (collected at Al Lith and Farasan Banks) to assess the likelihood that Symbiodiniaceae genotypes (represented in this study by ITS2 type profiles) associating with clams from the three most northern sites (Gulf of Aqaba, Duba, Thuwal) may be physically available for uptake by the clams at the two most southern sites (Al Lith, Farasan Banks). We searched for sequences that were present in the northern site clams and the southern site seawater samples, but not present in the southern site clams.

Our rationale was that while there would likely be a considerably larger richness of ITS2 sequences in the seawater samples compared to the clam samples, much of this richness would be representative of Symbiodiniaceae taxa potentially unable to form associations with *T. maxima* individuals (e.g., specialist free‐living taxa). We therefore constrained our search for sequences in the seawater samples to those that were known to be found in *T. maxima* individuals (from the northern sites). Finally, we were interested in finding evidence of Symbiodiniaceae diversity that the southern *T. maxima* individuals could potentially be associated with but had not (evidence of selectivity). As such, we further constrained our search to those sequences not found in the southern *T. maxima* samples.

Samples were distributed into three groups: clam samples from the three most northern sites, clam samples from the two most southern sites, and the two seawater samples. For each group, we generated a set of sequences that were found at least once in any of the group member samples (i.e., a set of present sequences; using the post‐MED abundance count tables output by SymPortal). We then performed union, intersection, and difference operations on these sets to determine unique or shared sequence members for every combination of the groups. The set operations were performed in Python using a bespoke script, and a three‐way Venn plot was generated using venn3 from the Python package matplotlib‐venn.

### 2.7 Availability of outputs and scripts used in this study

For outputs used in this study, and the results of the SIMPER analysis, please see Dryad submission https://doi.org/10.5061/dryad.k6djh9w50 and GitHub repository https://github.com/didillysquat/rossbach_2020. The Python scripts used in data processing and figure creation are also available at the GitHub repository.

## 3 RESULTS

### 3.1 *Tridacna maxima* ITS2 haplotypes from the Red Sea

To consolidate the current and previous efforts with regard to Symbiodiniaceae genotyping of *T. maxima* in the Red Sea, we compared the ITS2 sequences determined by Pappas et al., (2017; 202 sequences) and Weber (2009; 20 sequences) to our dataset. In the Pappas et al., dataset, after cropping and alignment, three haplotypes that showed congruence with the generated phylogenies represented 179 out of the 222 sequences (Figure 1b). The remaining 43 sequences were conservatively not considered biologically genuine as without access to sequence chromatograms their representation of sequencing artifacts cannot be discounted. In the ITS2 region of the three most abundant haplotypes, each exactly matched the A1 ITS2 sequence. The second and third most abundant haplotypes differed from the first by mutations in the ITS1 and 5.8S regions, respectively. Two distinct haplotypes were present in the Weber sequence collection. The most abundant haplotype was recovered from both sites sampled on the western Red Sea in Egypt (Hurghada and El Qeseir, Figure 1b), and one of the Gulf of Aqaba sites (Ras Nasrani). This haplotype was an exact match to one of the lesser abundant haplotypes sampled by Pappas et al., at the central, eastern Red Sea location. The second haplotype recovered by Weber was found in all but one of the specimens sampled at the second, more northern Gulf of Aqaba site (Dahab) and exactly matched the most abundant haplotype sampled by Pappas et al., (Thuwal, Figure 1b). One of the sequences in the Weber collection (from Dahab) had an ambiguous nucleotide call at the exact location of the mutation that differentiates the third major haplotype of the Pappas et al. collection. It is thus highly likely that all three major haplotypes sampled by Pappas et al. were recovered a decade earlier by Weber.

### 3.2 ITS2‐type profiles of *Tridacna maxima* across the Red Sea

**FIGURE 2:**
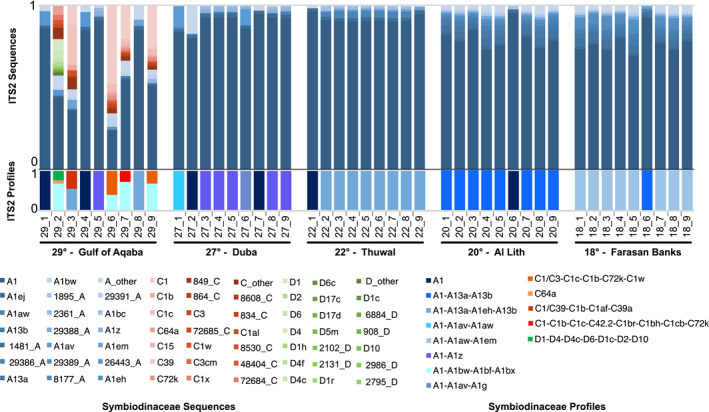
Symbiodiniaceae diversity of *Tridacna maxima* across the Red Sea. Genus‐annotated abundances of ITS2 sequences and predicted ITS2 type profiles (above and below, respectively) arranged by sampling site. For each recovered genus, the 20 most common post‐MED ITS2 sequences are plotted with remaining sequences binned into a single “other” category. Predicted profiles are plotted below the sequences. Blue: *Symbiodinium*; Orange: *Cladocopium*; Green: *Durusdinium*. Please refer to Figure S1 for a more thoroughly annotated version of this figure

**FIGURE 3:**
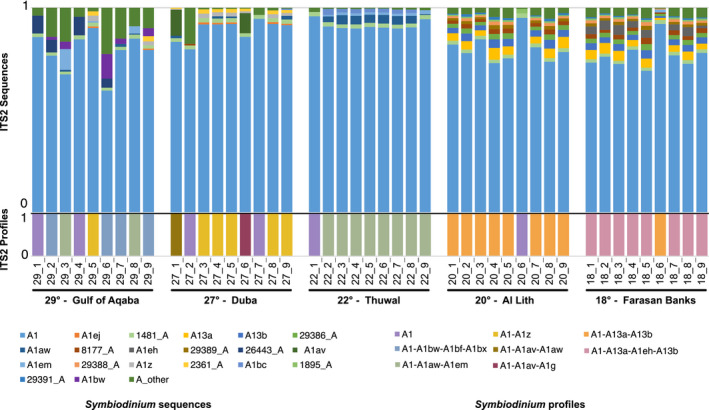
*Symbiodinium* diversity of *Tridacna maxima* across sites. Relative abundance of the 20 most common post‐MED ITS2 *Symbiodinium* sequences and predicted ITS2 type profiles (above and below, respectively) arranged by sampling site. Remaining sequences are binned into a single “other” category

Out of the 13 predicted distinct *T. maxima*‐associated ITS2 profiles predicted, the majority were *Symbiodinium* in origin (eight distinct profiles, recovered in 45 samples, representing 97.07% of the profile‐defining sequences), while the others were either *Cladocopium* (four distinct profiles, recovered in five samples, representing 2.63% of the profile‐defining sequences) or *Durusdinium* (recovered in one sample, representing 0.30% of the profile‐defining sequences). Most profiles were defined by 3 or more defining intragenomic variants (DIVs; 10/13 profiles) (Figures 2 and 3). While in general, a greater number of DIVs defining a profile is associated with an increased likelihood that the profile accurately represents a unique genotype, and we found six single‐DIV profile samples. These profiles had the A1 sequence predicted as the sole profile and likely represent the sampling of rarer Symbiodiniaceae genotypes (for further discussion, please see the Supplementary Results). Results of the SIMPER analysis showed that in all site comparisons, the A1 sequence accounted for the largest percentage of dissimilarity. The next most informative sequences were those DIVs defining the predominant ITS2 type profiles of the given sites being compared.

### 3.3 Higher Symbiodiniaceae diversity in the Gulf of Aqaba

All non‐*Symbiodinium* profiles were recovered from the Gulf of Aqaba (29°) where several clam samples harbored a mix of Symbiodiniaceae genera. Genera‐level profile diversity was therefore highest at this site (three genera) and absent at the four other sites (Figure 2). Clam‐associated *Cladocopium* and *Durusdinium* sequence diversity was also low outside of the Gulf of Aqaba with only one sample returning one *Cladocopium* sequence at a relative abundance of 0.00014 (no *Durusdinium* sequences were returned). While sequences from *Symbiodinium* were more abundant than those from *Cladocopium* and *Durusdinium* (76% ± 24% vs. 22% ± 23% and 3% ± 8%, respectively) in the Gulf of Aqaba, differences in ITS2 array copy number between different Symbiodiniaceae taxa, specifically at the genus level, preclude the accurate inference of the relative abundance of these Symbiodiniaceae genera at this site.

### 3.4 *Symbiodinium* diversity and profile distributions across the Red Sea

**FIGURE 4:**
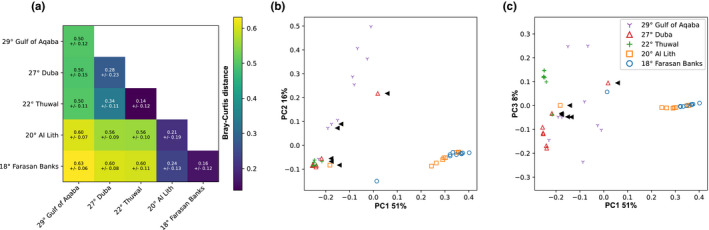
*Symbiodinium* diversity of *Tridacna maxima* across the Red Sea. (a) Heat map of between site, average sample Bray–Curtis distances. Colors represent the average distance. Both the average distance and the standard deviation are annotated for each pairwise comparison (above and below, respectively). For self‐comparisons (e.g., Duba‐Duba), distances represent the average between‐sample distance for the given site. (b, c) Principal coordinate analysis (PCoA) based on Bray–Curtis distances. PC1 vs. PC2 and PC1 vs. PC3, respectively. The PCoA shows the grouping of *T. maxima* host samples based on associated *Symbiodinium* sequences from the five sampling sites: in the Gulf of Aqaba—29° (purple/Y), Duba—27° (red/triangle), Thuwal—22° (green/+), Al Lith—20° (orange/square), and Farasan Banks—18° (blue/circle). Black arrows indicate samples with the single‐DIV profile of “A1”

Given the prominent association of *T. maxima* with *Symbiodinium*, we did a dedicated analysis to elucidate fine‐scale differences of association. Variation of between‐sample dissimilarities within sites was highest in the two northernmost sites, the Gulf of Aqaba (29°) and Duba (27°; Figure 4a). This observation, based on a sequence assemblage metric, is in concordance with the higher number of profiles predicted at the sites (five distinct profiles; Figure 3). The central (i.e., Thuwal, 22°) and the two southern sites (i.e., Al Lith, 20° and Farasan Banks, 19°) displayed the lowest assemblage variation and only two distinct profiles per site, with a notably high degree of profile homogeneity (at each site, eight out of the nine samples had the same single predicted profile; Figure 3).

Across the five sampled sites, three groups formed based on sequence assemblage dissimilarity (Figure 4a). The Gulf of Aqaba (29°) grouped separately from all other sites displaying the highest average between‐site sample variation (Figure 4b). The remaining four sites formed two groups, the first group consisting of the northern site at Duba (27°) and the central site at Thuwal (22°) and the second group with the two southern sites (i.e., Al Lith, 20° and Farasan Banks, 19°). The Gulf of Aqaba and the central/northern grouping were more similar to each other than to the southern grouping (Figure 4b,c).

Discounting the single‐DIV A1 profile occurrences, in general, a high site‐profile specificity was apparent, with a single specific profile dominant at each site (Figure 3). This finding is congruent with the significant PERMANOVA results returned from our analysis (pseudo‐*F* = 16.04, *p* < .001). However, in some cases, these dominant profiles were found at other sites at rarer abundances. Specifically, the A1‐A13a‐A13b profile dominant at Al Lith (20°) was recovered from a single sample in the Farasan Banks (19°), and the two profiles dominant at Thuwal (22°) and Duba (27°) (A1‐A1aw‐A1em and A1‐A1z, respectively) were recovered at the Gulf of Aqaba (29°). A reciprocal commonality was not recorded (i.e., the dominant profile at the Gulf of Aqaba, A1‐A1bw‐A1bf‐A1bx, was not recovered at Duba or Thuwal, and the dominant profile at Farasan Banks, A1‐A113a‐A1eh‐A13b, was not recovered at Al Lith).

### 3.5 Availability of northern clam‐associated Symbiodiniaceae genotypes to southern clams

We searched for sequences that were present in the northern site clams and the southern site seawater samples, but not present in the southern site clams. We found 18 ITS2 sequences that fit these criteria (Figure S2) from *Symbiodinium*, *Cladocopium,* and *Durusdinium*. Eight of the 18 sequences (A1z, A1g, C1, C3, D1, D4, D6, D4c) were DIVs from ITS2 type profiles associated with clams from either the Gulf of Aqaba or Duba.

Although not definitive proof, this result is highly suggestive of the fact that Symbiodiniaceae genotypes, in addition to those recovered in this study, are available for uptake by *T. maxima* at the southern sites, but that the southern clams are selectively associating with an alternative, relatively small proportion of the available diversity.

## 4 DISCUSSION

### 4.1 Characterization of *Tridacna maxima*‐Symbiodiniaceae associations

Here, we have leveraged intragenomic diversity of the ITS2 marker to generate fine‐scale delineations of *T. maxima*‐associated Symbiodiniaceae communities along 10° of latitude, spanning from the southern to the northern East coast of the Red Sea. In agreement with previous studies on Tridacninae‐Symbiodiniaceae associations from other regions, such as the Cook Islands and Papua New Guinea (Weber, 2009), as well as Indonesia (DeBoer et al., 2012), we found that Red Sea *T. maxima* clams harbor Symbiodiniaceae from three different genera, *Symbiodinium*, *Cladocopium,* and *Durusdinium*. Our results also show that *T. maxima* can establish symbiosis with all of these symbionts genera simultaneously, a finding that is consistent with previous reports from other regions. While *Symbiodinium* profiles were found in all *T. maxima* specimens investigated, only clams from the northernmost site in the Gulf of Aqaba (29°) also harbored symbionts with *Cladocopium* and *Durusdinium* profiles. A similar location dependency of the specific community composition of associated Symbiodiniaceae was previously reported for giant clams from the Coral Triangle (DeBoer et al., 2012) and the northern South China Sea (Lim et al., 2019), where differences were assumed to be mainly shaped by location of the habitat, specifically depending on the thermal and light regime (DeBoer et al., 2012; Lim et al., 2019), but also the host size (Lim et al., 2019).

Our finding, that *Symbiodinium* seems to be the dominant genus in Red Sea *T. maxima* clams, is consistent with the few available reports from the region. However, these previous studies, from the Egyptian, North‐western Red Sea coast and the Gulf of Aqaba (Weber, 2009), as well as from reefs in the central‐eastern Saudi Arabian Red Sea (Pappas et al., 2017), concluded that Red Sea giant clams associate exclusively with *Symbiodinium*. Yet, our results show that Red Sea *T. maxima* also associates with *Cladocopium* and *Durusdinium*. However, these genera appear to be less dominant than *Symbiodinium,* and our finding of these genera at a single site in the Gulf of Aqaba, coupled with Weber's finding of only *Symbiodinium* at two Gulf of Aqaba sites, suggests that their association with Tridacninae is rare and may be restricted to a small site‐specific distribution.

Our finding of a considerable diversity of *Symbiodinium* populations that have the A1 sequence as their most abundant ITS2 sequence is also in agreement with the multiple distinct ITS haplotypes previously reported from Weber and Pappas et al., (each of which had an exact match to the A1 ITS2 sequence). The resolution afforded by our intragenomic diversity‐defined ITS2 profiles and the full ITS region haplotypes may be similar. Of the 9 sites sampled by Pappas et al., a site referred to as “site 1” in their study was closest to our “Thuwal” site (presumably the same sampling site; although all sampling sites were within 28 km distance to our “Thuwal” site). Three approximately equally dominant haplotypes were recovered from this “site 1.” In contrast, we recovered only a single dominant ITS2 profile. However, the single‐DIV A1 profile recovered in one of our samples is likely symptomatic of the additional extant diversity at this site. As such, differences in the number of operational taxonomic units recovered may be largely due to sampling methodology (i.e., sampling depths, numbers of samples) rather than a difference in resolution. Finally, our findings of shared profiles between Thuwal (22°), Duba (27°), and the Gulf of Aqaba (29°) are also in agreement with the findings of identical ITS haplotypes recovered at Weber's sites in Egypt (North‐western Red Sea) and the Gulf of Aqaba, and Pappas et al.’s Thuwal sites in the central‐eastern Red Sea (as demonstrated by our reanalysis of available Symbiodiniaceae ITS haplotype data from the Red Sea; Figure 1b). Importantly, the fact that the ITS haplotype samplings were conducted approximately 8 years apart would indicate a significant temporal stability of these clam‐Symbiodiniaceae associations and corroborate previous notions of high symbiont fidelity (Howells et al., 2020; Terraneo et al., 2019), even through putative episodes of bleaching (Hume et al., 2020). However, and contrary to the temporal stability of such associations, we found largely distinct site associations, as discussed in the following.

### 4.2 Diversity and regional structuring of Symbiodiniaceae assemblages correlate with regional hydrographic gradients

The Red Sea displays distinct natural latitudinal gradients of temperature (Agulles et al., 2020; Chaidez et al., 2017), which are overall high and increase toward the South, and salinity (Arz et al., 2003; Ngugi et al., 2012), which is high in the North and shows a decrease toward lower latitudes. The antagonistic nature of these two gradients produces a high diversity of prevailing environmental conditions in Red Sea coral reefs, with a strong spatial variance, especially when comparing reefs from the North (i.e., cooler and more saline) to those in the South (i.e., warmer and less saline).

These pronounced latitudinal and environmental gradients have been shown to shape the genetic population structure of a number of marine species, resulting in a distinct genetic break of their populations at a latitude of approximately 19°N (Froukh & Kochzius, 2007; Giles et al., 2015; Nanninga et al., 2014; Shefer et al., 2004). Specifically, they have been reported to shape symbiont associations, for example, in *Porites* corals, where associated algal symbionts have been shown to shift from a *Cladocopium*‐ to a *Durusdinium*‐dominated community, along the North–South gradient of the Red Sea (Terraneo et al., 2019). Our results indicate a regional and site‐specific structuring of giant clam‐Symbiodiniaceae associations along the latitudinal environmental gradient in the Red Sea. At the regional level, we identified three groupings, based on sequence assemblage dissimilarity, reflecting the latitudinal North‐South gradient, as ITS2 assemblages from the Gulf of Aqaba (29°) and the central/northern grouping were more similar to each other than to the southern grouping. However, of particular note is the observed grouping of Duba (27°) and Thuwal (22°) in the northern and central Red Sea, respectively, despite the very large geographical distance (~700 km) between these two sites. However, the Symbiodiniaceae communities at Thuwal (22°) and Al Lith (20°) were considerably dissimilar despite the short geographic distance (~250 km) between these sites. This genetic structuring yet further supports the concept of a genetic discontinuity across Red Sea marine taxa at approximately 19–20°N (Froukh & Kochzius, 2007; Giles et al., 2015; Nanninga et al., 2014; Shefer et al., 2004).

Of the five sites sampled in this study, Symbiodiniaceae communities were more diverse in the northern sites (i.e., Gulf of Aqaba, 29° and Duba, 27°) than in the more southerly sites. Symbiodiniaceae communities in the Gulf of Aqaba, where *T. maxima* harbored Symbiodiniaceae from different genera, were particularly diverse (although see the *Symbiodinium*‐only recovery of Weber in the Gulf of Aqaba). These northern sites are characterized by the highest salinities (up to 40.5; Reiss & Hottinger, 2012), yet overall lowest sea surface temperatures in the Red Sea with a temperature maximum of about 27°C in the summer (Chaidez et al., 2017; Reiss & Hottinger, 2012). In addition, the northern Red Sea, and especially the Gulf of Aqaba, is characterized by a strong hydrographical seasonality (Badran, 2001; Manasrah et al., 2007), and particularly low water temperatures during the winter months (20°C; Reiss & Hottinger, 2012). Comparably extreme and fluctuating environmental conditions also exist in the Arabian Gulf, where waters are also hypersaline (>41.5; Yao & Johns, 2010) and organisms experience a pronounced seasonality, reflected in the extreme range of water temperatures between summer (35°C) and winter (15°C; Hume, D’Angelo, et al., 2018). In the Arabian Gulf, however, these extreme environmental conditions have been reported to lower the diversity of associated Symbiodiniaceae in different coral species (D’Angelo et al., 2015; Smith et al., 2017). While both are hypersaline and have a considerable seasonal fluctuation, the maximum and minimum temperatures of the Gulf of Aqaba are considerably less extreme than those in the Arabian Gulf. The lower maximum temperatures of these more northern Red Sea sites, relative both to the Arabian Gulf and the more southerly Red Sea sites, can be hypothesized, therefore, to be the most likely driver of Symbiodiniaceae assemblage diversity.

### 4.3 Panmictic distribution of *Tridacna maxima* suggests environmental—rather than host genotype‐driven assemblage structuring

The observed site‐specific structure of the Red Sea giant clam‐Symbiodiniaceae associations contrasts with the recently reported population structure of the *T. maxima* host. Current evidence points toward a genetic disparity between the Red Sea *T. maxima* clams and other populations from the West Indian Ocean (Fauvelot et al., 2020), but that the population inside the Red Sea is characterized by high gene flow among regions and panmixia (Lim et al., 2020). Yet, Lim et al., also observed a high level of host haplotypic diversity within the Red Sea population of *T. maxima* (i.e., a number of haplotypes at each site), which contrasts with the observed homogeneity of the associated Symbiodiniaceae assemblages that we identified here, especially in the more southern sites. In corals, fine‐scale resolutions of Symbiodiniaceae assemblages often strongly correlate to host genotype (Gardner et al., 2019; Howells et al., 2020; Hume, D’Angelo, et al., 2018; Hume et al., 2020). However, if the Red Sea *T. maxima* populations are assumed to be a single well‐connected, yet diverse population, this would suggest that the prevailing environmental conditions, which are also known to strongly influence coral‐Symbiodiniaceae associations (Hume et al., 2020; LaJeunesse et al., 2010; Oliver & Palumbi, 2009; Smith et al., 2020; Terraneo et al., 2019; Varasteh et al., 2017; Voolstra, Buitrago‐López, et al., 2020; Voolstra, Valenzuela, et al., 2020; Ziegler et al., 2015), are the driving forces of the observed structure. Whether this relatively high clam‐symbiont flexibility is a product of the location in which the algal symbionts reside within the host tissue (i.e., extracellularly in clams vs. intracellularly in corals) remains to be investigated.

### 4.4 Inter‐site flexibility of *Tridacna maxima*‐Symbiodiniaceae assemblages as a putative mechanism for niche adaptation

The finding of multiple *T. maxima* mtCOI marker haplotypes (distinct from those found in the wider Indian Ocean) at most of the Red Sea sites sampled would suggest that either one or multiple endemic panmictic populations of *T. maxima* are present along the latitude of the Red Sea. Given the taxonomically broad support of the 19° genetic discontinuity, it would seem exceptional that no such break is seen in *T. maxima* population(s). Either the host must be considered to possess an exceptional plasticity to survive in such a range of environments, or it must have some other mechanisms by which it is able to niche adapt. Given the diversity of recovered Symbiodiniaceae genotypes in this study, it would appear that *T. maxima‐*Symbiodiniaceae associations have a degree of flexibility and that there is a relatively high diversity of Symbiodiniaceae with which this host may associate. Our finding that *T. maxima* in the two most southern reefs associate with a relatively narrow diversity of Symbiodiniaceae, despite the presence of a much wider diversity that likely includes genotypes from the more northern sites, supports this notion of flexibility but also suggests a degree of selectivity by the clam hosts. Given that the high site fidelity seen in this study is most likely environmentally driven, flexibility and selectivity in these associations may offer a mechanism of adaptation for the host. In contrast to corals, where more fine‐scale resolutions increasingly reveal a relatively specific host genotype‐determined algal assemblage, such flexibility in clams represents, if confirmed, a mechanism conferring giant clams an additional resilience to warming. Indeed, although coral reefs in the Red Sea, particularly those in the southern Red Sea, have experienced intense warming‐induced bleaching in the past, such mass bleaching events have not been reported for Red Sea *T. maxima* populations (Lim et al., 2020). Testing this hypothesis would require experimental assessment of thermal performance of *T. maxima* under concurrent manipulation of their symbionts.

## CONFLICT OF INTEREST

The authors declare that the research was conducted in the absence of any commercial or financial relationships that could be construed as a potential conflict of interest.

## AUTHOR CONTRIBUTION

**Susann Rossbach:** Conceptualization (equal); Data curation (equal); Formal analysis (equal); Investigation (equal); Methodology (equal); Resources (equal); Visualization (equal); Writing‐original draft (equal); Writing‐review & editing (equal). **Benjamin CC Hume:** Data curation (equal); Formal analysis (equal); Investigation (equal); Methodology (equal); Software (equal); Supervision (equal); Visualization (equal); Writing‐original draft (equal); Writing‐review & editing (equal). **Anny Cardenas:** Data curation (equal); Formal analysis (equal); Methodology (equal); Resources (equal); Writing‐review & editing (equal). **Gabriela Perna:** Data curation (equal); Formal analysis (equal); Methodology (equal); Resources (equal); Writing‐review & editing (equal). **Christian R. Voolstra:** Conceptualization (equal); Formal analysis (equal); Investigation (equal); Methodology (equal); Project administration (equal); Resources (equal); Supervision (equal); Writing‐original draft (equal); Writing‐review & editing (equal). **Carlos Duarte:** Conceptualization (equal); Funding acquisition (equal); Project administration (equal); Resources (equal); Supervision (equal); Writing‐review & editing (equal).

## Supporting information

Fig S1‐S2

## Data Availability

Raw sequencing data of the *Tridacna maxima*‐Symbiodiniaceae communities, obtained for this, study are available under NCBI BioProject accession number #657469 (https://www.ncbi.nlm.nih.gov/bioproject/657469). All scripts that were used to create the distance ordination and related figures can be found in a GitHub repository under https://github.com/didillysquat/rossbach_2020. The SymPortal outputs and the multiple sequence alignment created are submitted to Dryad here: https://doi.org/10.5061/dryad.k6djh9w50
