# Supplementary material for: Flexibility in Red Sea Tridacna maxima‐Symbiodiniaceae associations supports environmental niche adaptation
Source: Ecol Evol. 2021 Mar 11;11(7):3393–406. doi: 10.1002/ece3.7299 (PMC8019035; doi:10.1002/ece3.7299)
Supplement: Supplementary file 1 — Fig S1‐S2 [file ECE3-11-3393-s001.docx]

**Flexibility in Red Sea *Tridacna maxima*-Symbiodiniaceae associations supports environmental niche adaptation**

**Supplementary Information**

Susann Rossbach^1†^, Benjamin C.C. Hume^2,3†*^, Anny Cárdenas^3^, Gabriela Perna^3^, Christian R. Voolstra^2,3^ and Carlos M. Duarte^1^

^1^ Biological and Environmental Science and Engineering Division, Red Sea Research Center (RSRC) and Computational Bioscience Research Center (CBRC), King Abdullah University of Science and Technology (KAUST), Thuwal, Kingdom of Saudi Arabia

^2^ Biological and Environmental Science and Engineering Division, Red Sea Research Center (RSRC), King Abdullah University of Science and Technology (KAUST), Thuwal, Kingdom of Saudi Arabia

^3^ Department of Biology, University of Konstanz, 78457 Konstanz, Germany

^†^ Authors with equal contribution

* Corresponding author: Benjamin C. C. Hume (benjamincchume@gmail.com)

**Running title:** Red Sea giant clam algal symbionts

**Supplementary Figures**

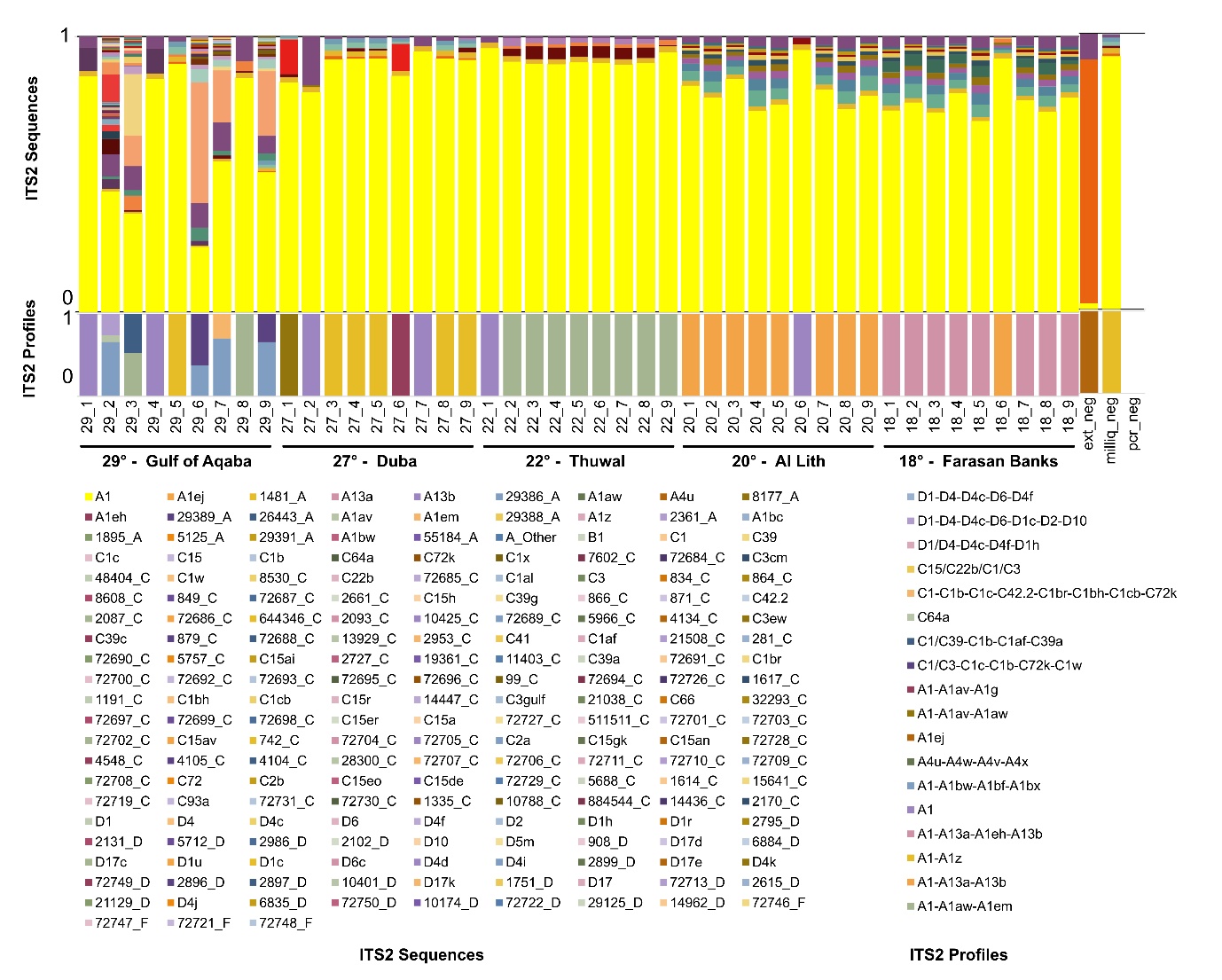


**Supplementary Figure S1.** Annotated abundances of ITS2 sequences and predicted ITS2 type profiles (above and below, respectively) arranged by sampling site and for the three negative controls (i.e. the lab and kit contaminants, including negative extraction and MilliQ; and the negative PCR). Predicted profiles are plotted below the sequences.
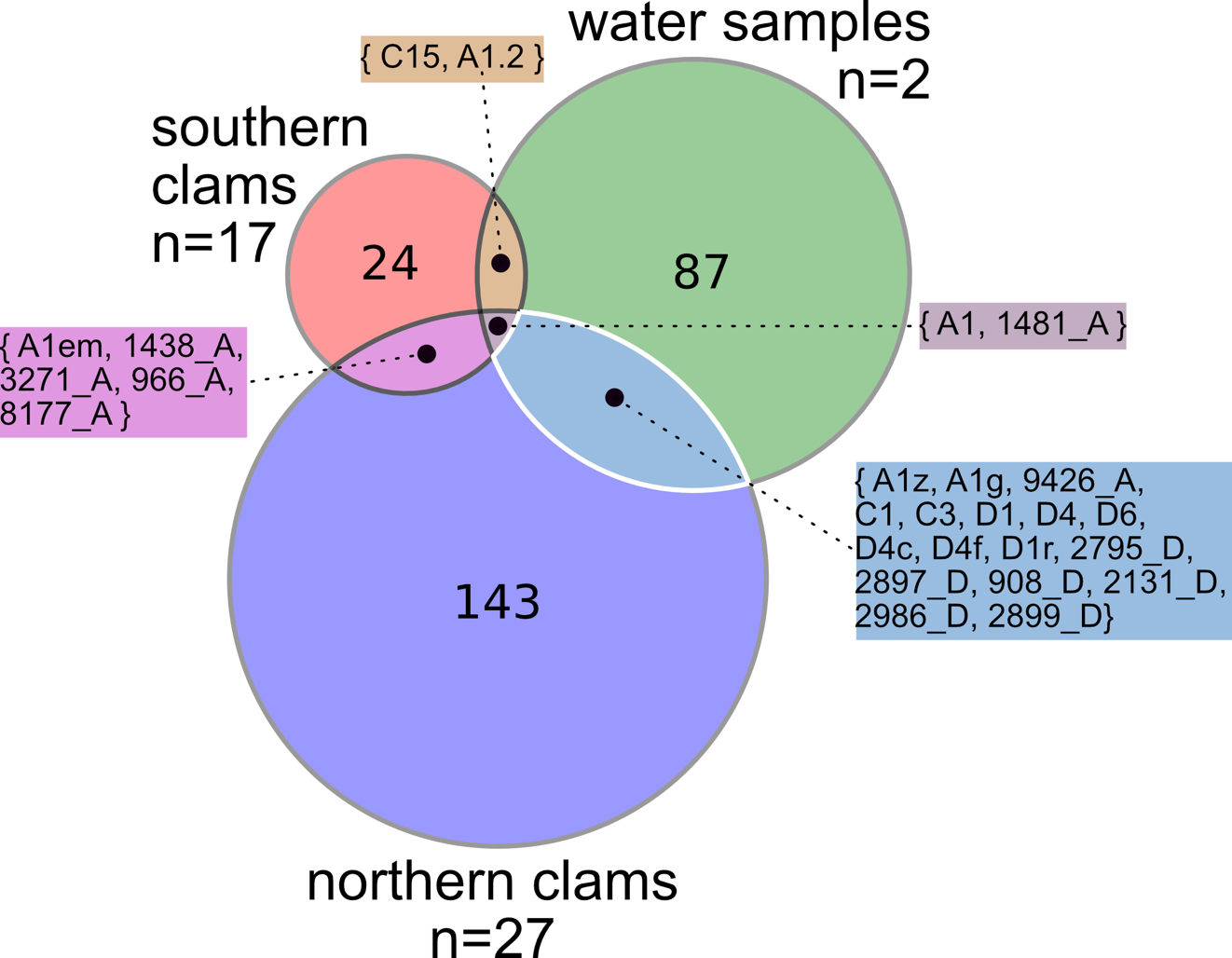


**Supplementary Figure S2.** The Venn diagram details the presence/absence of ITS2 sequences between the study’s samples grouped as ‘northern clams’ (all clam samples from the three most northern sites: Gulf of Aqaba, Duba, Thuwal), ‘southern clams’ (all clam samples from the two most southern sites: Al Lith, Farasan Banks) and ‘water samples’ (the two water samples collected at Al Lith and Farasan Banks). The numbers in the Venn segments refer to the number of ITS2 sequences. The ‘n=x’ refers to the number of samples contained in the given group. For the Venn segments representing unions of two or three groups, the sequences represented by those segments are detailed in the corresponding colored box. The white-outlined Venn segment, representing sequences found in the northern and seawater samples, but not the southern samples, as discussed in the main manuscript, is outlined in white.

**Supplementary Results**

This single DIV represents a case where the SymPortal algorithm has assigned a conservative profile that consists of only the most abundant sequence for the given sample, for the given genus/clade. This conservative prediction is a product of the fact that sets of the sequences found in one such sample, could not be found in a sufficient number of other samples to support the prediction of a more derived profile (i.e. one defined by a larger number of sequences; reciprocally, sets of samples used to define more derived profiles in other samples, were not found in these samples). These profiles most likely represent the sampling of rarer Symbiodiniaceae genotypes (sampled in insufficient quantity to enable a more derived profile to be assigned). A less parsimonious interpretation of these one DIV profiles (given the cutoffs implemented within the SymPortal search algorithms) would be that they are the due to a processing artefacts that resulted in a DIV that was present in the genotype, not being recovered.
